# Multifaced Nature of Yohimbine—A Promising Therapeutic Potential or a Risk?

**DOI:** 10.3390/ijms252312856

**Published:** 2024-11-29

**Authors:** Agnieszka Nowacka, Martyna Śniegocka, Maciej Śniegocki, Ewa Ziółkowska, Dominika Bożiłow, Wojciech Smuczyński

**Affiliations:** 1 Department of Neurosurgery, Nicolas Copernicus University in Toruń, Collegium Medicum in Bydgoszcz, ul. Curie Skłodowskiej 9, 85-094 Bydgoszcz, Poland; 2 Department of Anatomical, Histological, Forensic & Orthopedic Sciences, Section of Histology & Medical Embryology, Sapienza University of Rome, Via A. Scarpa, 14-16, 00161 Rome, Italy; 3 Department of Pediatrics, Washington University School of Medicine, St. Louis, MO 63110, USA; 4 Anaesthesiology and Intensive Care Clinical Ward, The 10th Military Research Hospital and Polyclinic, ul. Powstańców Warszawy 5, 85-681 Bydgoszcz, Poland; 5 Department of Physiotherapy, Nicolas Copernicus University in Toruń, Collegium Medicum in Bydgoszcz, ul. Techników 3, 85-801 Bydgoszcz, Poland

**Keywords:** yohimbine, indole alkaloid, α2-adrenergic receptors

## Abstract

A natural compound derived from the Pausinystalia yohimbe tree—yohimbine, has a rich history of use in traditional medicine and is currently being explored for its potential therapeutic applications. This indole alkaloid primarily acts as an antagonist of α2-adrenergic receptors. Initially recognized for its purported aphrodisiac properties, yohimbine has been investigated for a wide range of applications, including sports or the treatment of erectile dysfunction and metabolic disorders. However, toxicological concerns exist, particularly at higher doses. Ongoing researches help to fully assess yohimbine’s efficacy and safety profile and to explore strategies for enhancing its bioavailability and reducing toxicity. This review examines the multifaceted nature of yohimbine, delving into both its promising therapeutic potential and the associated risks.

## 1. Introduction

Yohimbine, an indole alkaloid obtained from the bark of the Pausinystalia yohimbe tree [1], has a lengthy and multifaceted history of being employed in traditional medicine and, more recently, as a potential therapeutic agent. Initially recognized for its purported aphrodisiac properties [2], yohimbine has been investigated for a wide range of applications. However, alongside its potential benefits, concerns regarding its safety and potential adverse effects have also emerged. This review aims to explore the multifaceted nature of yohimbine, examining both its promising therapeutic potential and the potential risks associated with its use.

## 2. Yohimbine Chemistry

**Figure 1:**
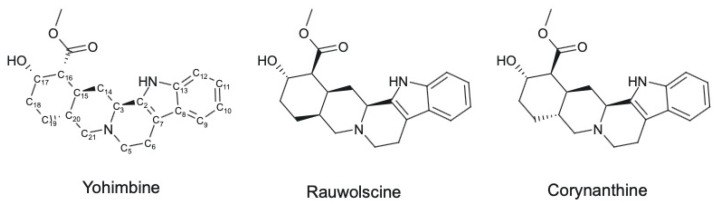
Yohimbine, rauwolscine and corynanthine—molecular structure.

Yohimbine—C_21_H_26_N_2_O_3_ (Figure 1) is a naturally occurring indole alkaloid primarily derived from the bark of the Pausinystalia yohimbe tree, indigenous to West Africa [3]. It is also found in other plant species like Pausinystalia yohimbe [4], Rauvolfia serpentina [4], Rauvolfia verticillata [5], Rauvolfia tetraphylla [6], Tribulus terrestris [7], Nicotiana tabacum [7], Peganum harmala [7], Rauvolfia vomitoria [8]. Yohimbine exists as a colorless, weakly basic compound with a pKa value between 6 and 7.5 [3]. It exhibits high solubility in alcohol and chloroform while being sparingly soluble in water and diethyl ether [3]. Yohimbine’s melting point is approximately 234 °C, and it decomposes at around 302 °C [3].

Yohimbine is a pentacyclic monoterpenoid indole alkaloid with a molecular weight of 354.44 g/mol. The core structure of yohimbine consists of a 17-α-hydroxyyohimban-16-α-carboxylic acid methyl ester, which is biosynthetically derived from the precursors tryptophan and the secoiridoid monoterpene secologanin [9]. This intricate chemical structure, featuring five chiral centers and two nitrogen atoms, contributes to the diversity of yohimbine stereoisomers, such as rauwolscine (Figure 1) and corynanthine (Figure 1), each exhibiting subtle differences in their physical properties and interactions with various receptors.

In recent years, the therapeutic potential of natural indole alkaloids, including yohimbine, has gained significant attention in both preclinical and clinical studies [10,11,12]. This growing interest reflects the broader recognition of the value of natural compounds as a rich source of novel lead molecules and the ongoing efforts to explore their diverse pharmacological applications, particularly in the field of oncology.

Structurally, yohimbine features a pentacyclic ring system with five chiral carbon atoms (C3, C15, C16, C17, and C20) and two nitrogen atoms [13]. This complex structure gives rise to several diastereomers, separable through chromatographic techniques [14]. The existence of multiple chiral centers contributes to the diversity of yohimbine stereoisomers, including rauwolscine and corynanthine, which exhibit subtle differences in their physical properties and interactions with dopamine/serotonin receptors. Yohimbine’s chemical structure shares similarities with reserpine but with different stereochemistry [3].

Biosynthetically, yohimbine originates from tryptophan and the secoiridoid monoterpene secologanin [9,15]. These precursors contribute to the formation of the pentacyclic monoterpenoid indole alkaloid structure [12]. The basic moiety in yohimbine arises from the indole moiety rather than the tertiary amine group [16].

The synthesis of yohimbine is complex due to the intricate formation of the pentacyclic rings and the presence of five chiral centers [17]. Several synthetic strategies have been developed, often involving the construction of a DE-ring system followed by C-ring formation or, alternatively, building an ABC-ring system and subsequently annulating a DE-ring [18]. The first reported synthesis of yohimbine yielded a racemic mixture through a series of steps to construct the DE trans group found in naturally occurring pentacyclic indole derivatives [19]. More recent synthetic approaches have focused on enantioselective synthesis, aiming to produce specific stereoisomers of yohimbine with higher yields [20,21].

## 3. Pharmacological Properties

**Figure 2:**
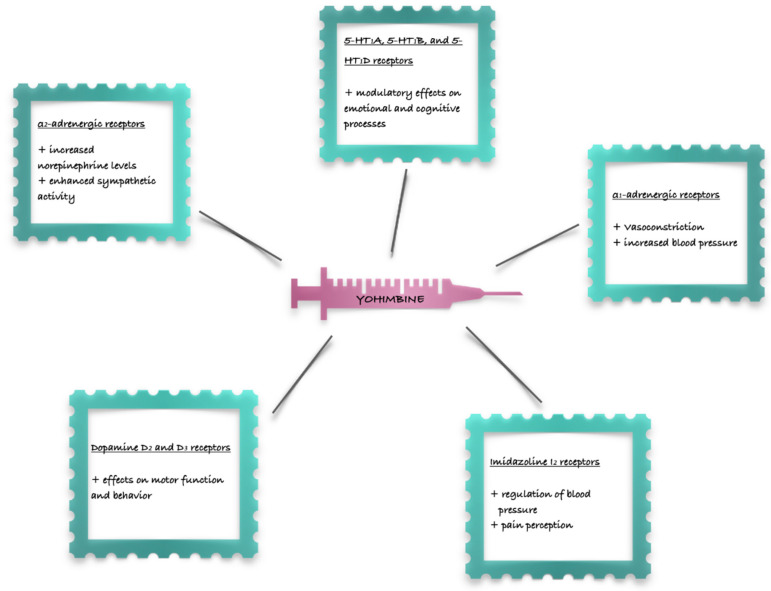
Yohimbine and its interaction with receptors.

Yohimbine exhibits a broad spectrum of pharmacological activities, primarily mediated through its interactions with various receptor systems. Its primary pharmacological action is as a selective α_2_-adrenergic receptor antagonist [22]. It exhibits a higher affinity for the presynaptic α_2_-adrenergic receptors compared to the postsynaptic ones. By blocking these receptors, yohimbine inhibits the negative feedback mechanism that normally suppresses norepinephrine release (Figure 2). This leads to increased norepinephrine levels in the synaptic cleft, resulting in enhanced sympathetic activity.

Beyond its α_2_-adrenergic antagonism, yohimbine interacts with several other receptors (Figure 2), albeit with lower affinity. These include:

1. 5-HT_1_A, 5-HT_1_B, and 5-HT_1_D receptors: Yohimbine shows moderate affinity for these serotonin receptors, which may contribute to some of its effects on mood and anxiety [23]. This interaction with serotonin receptors suggests that yohimbine may also have modulatory effects on emotional and cognitive processes.
2. α_1_-adrenergic receptors: While primarily an α_2_-antagonist, yohimbine can also act as an α_1_-agonist at higher concentrations, potentially leading to vasoconstriction and increased blood pressure [23]. This dual action on adrenergic receptors can result in complex physiological responses.
3. Dopamine D_2_ and D_3_ receptors: Yohimbine exhibits some affinity for these dopamine receptors, although its effects on dopaminergic pathways are less pronounced compared to its adrenergic actions [23]. The interaction with dopamine receptors may contribute to some of yohimbine’s effects on motor function and behavior.
4. Imidazoline I_2_ receptors: Yohimbine can also bind to these receptors, which are involved in the regulation of blood pressure and pain perception.

In addition to its other pharmacological properties, yohimbine has been shown to exhibit endothelin-like activity, which can affect nitric oxide (NO) production and vascular function. This endothelin-like effect may have implications for regulating blood flow and pressure [24]. Furthermore, yohimbine has been observed to induce anxiety-like behaviors through its actions on the noradrenergic pathway, stimulating the HPA (hypothalamic-pituitary-adrenal) axis [25] and leading to increased release of stress hormones such as cortisol. This anxiogenic property of yohimbine is an important consideration, given its potential therapeutic applications. Specifically, the endothelin-like actions of yohimbine may influence vascular tone and blood pressure regulation, while its stimulation of the HPA axis can result in heightened anxiety and stress responses. These multifaceted effects of yohimbine on the cardiovascular and neurological systems warrant careful consideration when evaluating its therapeutic potential, especially in the context of brain tumor treatment, where modulating vascular function and managing stress-related side effects may be critical factors.

The diverse interactions of yohimbine with various receptors contribute to its complex pharmacological profile and diverse physiological effects. Yohimbine has been shown to elevate blood levels of the catecholamines epinephrine and norepinephrine in healthy individuals, leading to increased heart rate, systolic blood pressure, and heightened alertness [26]. This elevation in catecholamines can have a wide-ranging impact on the cardiovascular system and overall physiological arousal. Additionally, yohimbine can enhance localized blood flow, potentially by constricting splanchnic vessels and redirecting blood to skeletal muscle [27]. This altered blood flow may have implications for anaerobic exercise performance by modulating skeletal muscle metabolism and lactic acid processing [28], although further research is needed to fully elucidate its impact. The multifaceted pharmacological actions of yohimbine also underscore the potential for side effects and drug interactions, particularly regarding its combined effects on adrenergic and serotonergic receptors, which can influence blood pressure, mood, and sexual function.

## 4. Dosing and Administration of Yohimbine

The administration and dosing of yohimbine depend on the specific condition being treated and the formulation used. Historically, yohimbine hydrochloride has been the most common medicinal form [13].

Yohimbine demonstrates rapid oral absorption, with an absorption half-life of approximately 10 min, and efficient excretion, characterized by an elimination half-life of around 30 min [22]. Therapeutic blood concentrations of yohimbine typically fall within the range of 50 to 300 ng/mL [1], with dosages ranging from 5–10 mg taken two to three times daily [13,29]. However, the optimal dose and frequency can vary based on individual patient factors and therapeutic objectives. For instance, some studies on the treatment of erectile dysfunction have utilized higher doses, up to 18 mg, with tailored adjustments based on patient response [13].

The oral bioavailability of yohimbine in humans exhibits substantial variability, ranging from a low of 10% up to a high of 90% [30]. This wide range indicates that the proportion of yohimbine reaching systemic circulation after oral administration can differ significantly between individuals. It is important to note that yohimbine’s bioavailability can be significantly influenced by various factors, such as food intake and individual metabolic differences.

Additionally, intramuscular and intravenous formulations of yohimbine have been developed and studied, particularly in preclinical and animal research models [31,32]. The use of parenteral routes of administration may increase the reliability and consistency of yohimbine delivery compared to oral dosing, although there are inherent practical and safety considerations to account for in a clinical setting.

The pharmacokinetics of yohimbine can be quite variable, with factors like stomach pH, gut microbiome, and hepatic enzyme activity all playing a role in its absorption, distribution, and clearance [33,34,35,36]. Furthermore, the purity and standardization of yohimbine supplements can vary considerably between different manufacturers and product batches. This lack of consistency in the composition of yohimbine supplements, particularly with regard to the presence and concentration of the active alkaloid, emphasizes the importance of selecting reputable, high-quality sources. Some supplements may contain yohimbe bark extract rather than pure yohimbine hydrochloride, and the alkaloid content in these extracts can fluctuate widely. This inconsistency poses significant challenges in determining accurate and reliable dosing, which in turn increases the risk of adverse effects for the end user.

While yohimbine can be well-tolerated at appropriate doses, it is essential to be aware of its potential toxicological aspects. Several factors influence the likelihood and severity of adverse effects, including dosage, individual sensitivity, and co-existing medical conditions.

Common side effects reported at therapeutic doses include anxiety, nervousness, insomnia, increased heart rate, and elevated blood pressure [1,37,38,39,40,41,42]. These effects are often dose-dependent and related to yohimbine’s sympathomimetic actions. Gastrointestinal issues like nausea and vomiting have also been reported [38,39,41,42].

At higher doses or in susceptible individuals, more severe adverse effects can occur. These may include cardiac arrhythmias, myocardial infarction, seizures, and renal failure [1,13,39,40,41,42]. Yohimbine can also exacerbate pre-existing psychiatric conditions like agitation, anxiety and panic disorders [13,37,43].

Drug interactions are another important consideration. Yohimbine can interact with medications that affect the adrenergic and serotonergic systems, such as antidepressants, antihypertensives, and stimulants [38,44]. Concurrent use with these medications may potentiate or alter yohimbine’s effects, increasing the risk of adverse events.

Due to the potential for a range of side effects and the possibility of interactions with other medications, yohimbine should be used with great caution and under the close supervision of a healthcare provider. Close and ongoing monitoring of blood pressure, heart rate, and other vital signs is strongly recommended, especially during the initial treatment period and when adjusting the dosage. Patients with pre-existing cardiovascular, neurological, or psychiatric conditions should exercise particular caution and thoroughly inform their healthcare provider about their full medical history before initiating any treatment regimen involving yohimbine. The complex and multifaceted pharmacological profile of yohimbine warrants a careful, individualized approach to its use and administration.

In their study, Mueller-Schoell et al. [45] examined yohimbine intoxication in four individuals who ingested a yohimbine-containing drug powder. It revealed significant variability in blood concentrations, ranging from 249 to 5631 ng/mL, a 22-fold difference despite a presumed uniform dose of 5 g. This variability was linked to the individuals’ cytochrome P450 2D6 (CYP2D6) phenotypes, with decreased CYP2D6 activity increasing the risk of toxic yohimbine levels. All four patients were identified as phenotypic CYP2D6 intermediate metabolizers, contributing to their reduced metabolic activity and higher yohimbine concentrations. Using nonlinear mixed-effects modeling, individual yohimbine clearances and CYP2D6 phenotypes were estimated based on observed concentrations. The model predicted clearances greater than 3000 mL/min for extensive metabolizers and below 100 mL/min for poor metabolizers. Incorporating CYP2D6 activity as a covariate significantly reduced interindividual variability in yohimbine clearance. Besides limitations, including the unknown purity of the ingested yohimbine and the lack of CYP2D6 genotype data for validation, these findings suggest that understanding a patient’s CYP2D6 metabolic activity and phenotyping patients for CYP2D6 activity before initiating yohimbine treatment might be crucial for determining safe doses and minimizing overdose risk.

## 5. Metabolism

Yohimbine is absorbed relatively quickly after oral ingestion, with a half-life of 7–11 min. Peak plasma concentrations are reached within 45–60 min. However, the amount of yohimbine that actually reaches systemic circulation varies greatly between individuals, with bioavailability ranging from 10% to 90% [3,30]. This wide range suggests significant individual differences in first-pass metabolism and other factors affecting absorption.

The metabolism of yohimbine involves several pathways and enzymes, which contribute to its variable pharmacokinetic profile. It undergoes extensive first-pass metabolism in the liver [46]. A significant portion of the drug is metabolized before reaching the systemic circulation, primarily through two pathways: oxidation to the pharmacologically active metabolite 11-hydroxy-yohimbine and a smaller percentage being converted to 10-OH-yohimbine [46].

The major metabolic pathway involves hepatic oxidation, primarily mediated by cytochrome P450 enzymes, specifically CYP2D6 and CYP3A4 [3,44]. CYP2D6 plays a crucial role in the 11-hydroxylation of yohimbine, forming 11-hydroxyyohimbine, a pharmacologically active metabolite [3]. Genetic polymorphisms in CYP2D6 can significantly influence yohimbine metabolism, leading to substantial interindividual variability in its clearance and clinical effects [3,44]. Individuals with certain CYP2D6 genotypes may exhibit impaired metabolism, resulting in higher plasma concentrations and an increased risk of adverse effects.

Other metabolic pathways include oxidation to 10-hydroxyyohimbine and other minor metabolites [3]. The kidneys excrete yohimbine and its metabolites, with less than 1% of the administered dose found unchanged in the urine after 24 h [47]. Yohimbine has a relatively short half-life of less than 1 h, indicating rapid clearance from the plasma [3].

The complex interplay of these metabolic pathways, coupled with the influence of genetic polymorphisms and other individual factors, contributes to the wide variability observed in yohimbine’s pharmacokinetics and clinical responses. Understanding these metabolic processes is crucial for optimizing dosing strategies and minimizing the risk of adverse events.

## 6. Erectile Dysfunction

Yohimbine has a long and complex history in the treatment of erectile dysfunction (ED), with ongoing discussions and debates surrounding its efficacy and role in contemporary management [48,49]. Its proposed mechanism of action involves the antagonism of α_2_-adrenergic receptors, leading to increased noradrenaline levels and subsequent vasodilation in the penile tissue [48]. This vasodilatory effect can theoretically facilitate improved blood flow, contributing to the ability to achieve and maintain an erection.

According to a study by Rupero et al. [50], yohimbine has demonstrated potential in the treatment of erectile dysfunction through its effective binding to hemoglobin, as evidenced by spectroscopic and molecular-docking analyses. The researchers found that yohimbine exhibits a high binding affinity to hemoglobin, with a value of 10^5^ M^−1^ and a 1:1 stoichiometry, indicating a strong interaction between the two. The spontaneous and entropy-driven nature of this protein-ligand association suggests that yohimbine’s mechanism of action in treating ED may involve hydrophobic forces and hydrogen bonding, which could contribute to its therapeutic efficacy in this context.

Several studies have demonstrated modest improvements in erectile function with the use of yohimbine, particularly in patients with mild to moderate ED [51]. A review of seven controlled studies indicated that yohimbine could be an effective therapy, with one study showing that 34% of patients experienced partial improvement and 20% reported full and sustained erections, compared to only 7% in the placebo group [52]. Additionally, a meta-analysis has demonstrated the superiority of yohimbine over placebo in the treatment of ED [49]. However, the overall evidence supporting its widespread use is considered limited, with some studies yielding mixed results [48,53]. The efficacy of yohimbine may also vary depending on the underlying cause of ED, with some evidence suggesting it may be more beneficial in psychogenic ED compared to organic causes.

It is important to note that yohimbine is not considered a first-line treatment for ED. Phosphodiesterase-5 inhibitors, such as sildenafil and tadalafil, are generally preferred due to their higher and more consistent efficacy, as well as their better-established safety profiles [54,55]. Yohimbine may be considered as an adjunctive therapy or in cases where PDE5 inhibitors are contraindicated or ineffective.

Yohimbine has shown promise as a standalone treatment or as part of combination therapies for addressing erectile dysfunction and low testosterone levels. A study by Kuchakulla et al. [56] found that yohimbine is a key active ingredient in many over-the-counter supplements marketed for improving male sexual and reproductive health. The researchers examined the efficacy of yohimbine and other common supplement components, such as L-arginine and ginseng, in boosting testosterone production and alleviating erectile dysfunction. Their findings suggest that the combination of yohimbine with these other natural ingredients can provide a multi-pronged approach to managing these common male health concerns. While more research is needed to fully understand the optimal dosing and formulations, this evidence indicates that yohimbine-containing supplements may offer a viable option for men seeking to address issues related to sexual function and testosterone deficiency.

Furthermore, the use of yohimbine in the treatment of ED is not without limitations. Its potential for side effects, including anxiety, nervousness, and increased blood pressure, can be problematic for some individuals [49]. Drug interactions are also a concern, particularly with medications affecting the adrenergic system. Careful patient selection and individualized dosing strategies are crucial to maximize the potential benefits and minimize the risks associated with yohimbine therapy.

## 7. Body Weight Reduction

Yohimbine has been investigated as a potential adjunct in weight management strategies due to its purported effects on metabolism and lipolysis. Its proposed mechanism of action involves antagonizing α_2_-adrenergic receptors, which are involved in regulating fat breakdown [3]. By blocking these receptors, yohimbine may increase noradrenaline release, thereby stimulating lipolysis and potentially promoting fat loss.

Some studies have demonstrated modest weight reduction outcomes with yohimbine supplementation, particularly when combined with exercise and dietary modifications. Iciek et al. [57] investigated the effects of yohimbine on oxidative stress and cysteine metabolism in the livers of rats fed a high-fat diet. Yohimbine was administered at doses of 2 mg/kg and 5 mg/kg for 30 days to HFD-induced obese rats. The HFD led to decreased hepatic levels of cysteine and sulfane sulfur while sulfate levels and lipid peroxidation increased. Yohimbine treatment at 5 mg/kg restored sulfate levels to control values, increased rhodanese expression and reduced lipid peroxidation, indicating a decrease in oxidative stress. However, it did not significantly affect levels of low molecular weight thiols or the expression of enzymes responsible for H2S synthesis. The study concludes that HFD-induced obesity disrupts cysteine metabolism and increases oxidative stress in the liver and that yohimbine, particularly at a higher dose, can partially alleviate these disturbances by restoring sulfate levels and reducing oxidative stress, though its overall impact on cysteine metabolism was limited. These findings suggest yohimbine’s potential as a therapeutic agent for managing obesity-related oxidative stress and metabolic disturbances.

Kotańska et al. [58] investigated the weight-reducing effects of yohimbine, an α_2_-adrenoceptor antagonist, in normal C57BL6 mice and obese, leptin-deficient ob/ob mice. Their findings revealed a key role for leptin in mediating yohimbine’s effects on body weight. In normal C57BL6 mice, yohimbine administration led to a reduction in body weight. This effect is attributed to yohimbine’s blockade of α_2_-adrenoceptors, which subsequently increases noradrenaline release and stimulates β_3_-adrenoceptors. β_3_-adrenoceptors are known to play a role in energy expenditure and lipolysis, thus contributing to weight loss. However, in obese, leptin-deficient ob/ob mice, yohimbine did not produce any significant weight reduction. This difference highlights the importance of leptin in yohimbine’s weight-reducing mechanism. Obese mice lack functional leptin, a hormone crucial for regulating appetite and energy balance. The absence of leptin, along with impaired α_2_ and β_3_-adrenoceptor function in these mice, likely renders yohimbine ineffective for weight loss. Interestingly, despite the lack of weight reduction in obese mice, yohimbine did improve their lipid and carbohydrate profiles. This suggests that yohimbine’s metabolic benefits may be independent of its weight-reducing effects and potentially mediated through different mechanisms. The authors propose that yohimbine’s blockade of α_1_-adrenoceptors, which occurs at higher doses, may contribute to these improvements in lipid and carbohydrate metabolism. These findings have important implications for understanding yohimbine’s therapeutic potential. While its weight-reducing effects appear to be dependent on leptin signaling and functional β_3_-adrenoceptors, its metabolic benefits may extend to individuals with leptin deficiency or impaired adrenoceptor function. The study suggests that non-selective α-adrenoceptor antagonists like yohimbine may offer therapeutic benefits for treating disrupted lipid-carbohydrate homeostasis, even in the absence of significant weight loss. Further research is needed to fully elucidate the mechanisms underlying these effects and to explore the potential clinical applications of yohimbine in managing metabolic disorders.

Studies by Dudek et al. [59] have shown that yohimbine is a non-selective antagonist of α2-adrenoceptors, acting primarily through α2A-adrenoceptors. At higher doses, it also acts as an α1-adrenoceptor antagonist. By blocking presynaptic α2A-adrenoceptors, yohimbine increases noradrenaline release, which then stimulates β-adrenoceptors and, to a lesser extent, α2B- and α1-adrenoceptors. These actions contribute to yohimbine’s weight-reducing and anti-obesity effects, as demonstrated in studies on diet-induced obesity in rats. Chronic administration of yohimbine has also been shown to improve impaired lipid profiles and lower glucose levels, likely due to its blockade of α1-adrenoceptors. Furthermore, the blockade of these adrenoceptors by yohimbine can lead to a reduction in sympathetic nervous system activity, which may have additional beneficial effects on metabolic parameters and overall weight management.

## 8. Anti-Inflammatory Effects

Yohimbine, beyond its recognized role as an α_2_-adrenergic receptor antagonist, exhibits promising anti-inflammatory properties through various interconnected mechanisms. While research continues to unravel the full complexity of these interactions, several key pathways have emerged:

1. Modulation of α_2_-Adrenergic Receptor Activity and Tyrosine Hydroxylase: Yohimbine’s anti-inflammatory effects also extend to modulating α_2_-adrenergic receptor activity. Inhibiting these receptors can upregulate tyrosine hydroxylase (TH), the enzyme responsible for catecholamine synthesis, including noradrenaline [3]. While the precise link between TH upregulation and anti-inflammatory action remains to be fully elucidated, evidence suggests a potential involvement of cAMP-mediated pathways. This intricate interplay between α_2_-adrenergic receptors, TH, and cAMP warrants further investigation to clarify its role in yohimbine’s anti-inflammatory effects.
2. Inhibition of Pro-inflammatory Cytokines and Modulation of Antioxidant States: Yohimbine has demonstrated efficacy in inhibiting pro-inflammatory cytokines and modulating antioxidant states, particularly in the context of arthritis [60]. Studies have shown a significant reduction in the expression of COX-2, TNF-α, and NF-κB, alongside decreased levels of ESR, WBC, and C-reactive protein in arthritic rats treated with yohimbine. These findings underscore yohimbine’s potential to ameliorate inflammation and oxidative stress in arthritic conditions. Moreover, yohimbine has shown protective effects against renal ischemia/reperfusion-induced acute inflammation [3], further broadening its potential therapeutic applications. A central mechanism of yohimbine’s anti-inflammatory action involves suppressing the NF-κB pathway [61]. NF-κB, a crucial transcription factor, orchestrates the expression of numerous pro-inflammatory cytokines, including IL-1β and IL-6. By inhibiting NF-κB activation, yohimbine effectively reduces the production of these inflammatory mediators. This inhibitory effect has been demonstrated in various experimental models. For instance, Ou et al. showed that yohimbine mitigated IL-1β or noradrenaline-induced IL-6 upregulation and cartilage destruction in condylar processes by suppressing the NF-κB pathway both in vitro and in vivo [61]. This suggests that yohimbine’s targeting of the NF-κB pathway may have therapeutic implications for inflammatory joint conditions.
3. Renoprotective Effects and Synergistic Action with Berberine: Recent research has highlighted yohimbine’s renoprotective effects in LPS-induced acute kidney injury [62]. Shimokawa et al. demonstrated that yohimbine ameliorates kidney damage and LPS-induced hypotension by suppressing cytokine mRNA, iNOS, and NF-κB activation. Furthermore, yohimbine enhances ERK and CREB phosphorylation, promoting IL-10 expression, an anti-inflammatory cytokine. Interestingly, combining yohimbine with berberine amplifies the therapeutic effect against LPS-induced bacteremia by synergistically inhibiting JNK, ERK, NF-κB, and other pathways through IL-10 upregulation. This synergistic action suggests potential therapeutic strategies for sepsis and other inflammatory conditions.

Yohimbine, in the study by Chayka et al. [63], plays a crucial role as a foundational compound for developing novel analogs with improved selectivity for treating inflammatory disorders and sepsis. While yohimbine’s nonselective antagonism of adrenoceptors limits its clinical use due to potential side effects, it serves as a basis for developing more selective agents. Researchers focused on amino esters of yohimbic acid, aiming to enhance selectivity for the ADRA2A receptor. Compound 4n, a novel analog, demonstrated significantly improved selectivity indices compared to yohimbine, with a six-fold increase in ADRA1A/ADRA2A selectivity and a 25-fold increase in ADRA2B/ADRA2A selectivity. This comparison highlights yohimbine’s limitations and the potential of its analogs for targeted therapeutic interventions. Furthermore, 4n exhibited high plasma and microsomal stability, suggesting sustained bloodstream effectiveness but moderate-to-low membrane permeability, potentially limiting its ability to cross the blood-brain barrier. Importantly, 4n showed negligible toxicity in non-tumor normal human dermal fibroblasts. These findings position 4n as a promising selective ADRA2A antagonist, offering a valuable tool for studying adrenoceptor subtypes in inflammatory disorders and sepsis and a novel preclinical candidate for ADRA2A-mediated pathologies.

Sharma et al. [64] investigated the anti-fibrotic and anti-inflammatory effects of yohimbine hydrochloride (YHC) in liver models. Their study demonstrated that YHC effectively reduced the expression of crucial inflammatory and fibrotic markers in both human liver sinusoidal endothelial cells and hepatic stellate cells. This suggests a protective effect against liver damage induced by agents such as lipopolysaccharide and transforming growth factor-beta, both known contributors to liver inflammation and fibrosis. The reduction in these markers indicates YHC’s potential to interrupt the fibrotic cascade and mitigate inflammatory responses within the liver. Furthermore, YHC was shown to decrease oxidative stress levels in HepG2 cells, a human liver cancer cell line commonly used to study liver function. This finding suggests that YHC may protect against oxidative damage, a key factor in the pathogenesis of liver diseases. While the specific mechanisms underlying this antioxidant effect were not fully elucidated, they add another layer to YHC’s potential therapeutic benefits in liver disease. In vivo experiments using a rat model of thioacetamide-induced liver injury further supported the protective effects of YHC. YHC treatment significantly improved survival rates in these rats, demonstrating its potential to translate in vivo and offering hope for its therapeutic application in liver diseases. Mechanistically, Sharma et al. found that YHC modulates the JNK/Wnt/β-catenin signaling pathway, a crucial pathway involved in liver inflammation and fibrosis. YHC reduced the nuclear translocation of β-catenin, a key downstream effector of the Wnt pathway. By inhibiting β-catenin’s activity, YHC likely disrupts the downstream signaling cascade that promotes inflammation and fibrosis. This mechanistic insight provides a potential explanation for YHC’s observed anti-fibrotic and anti-inflammatory effects. These findings collectively suggest that yohimbine, specifically in the form of YHC, holds promise as a potential therapeutic agent for liver fibrosis.

## 9. Anti-Cancer Effects

Elevated levels of catecholamines (noradrenaline and adrenaline), associated with activation of the sympathetic nervous system and the stress response, are linked to increased cancer risk and poorer prognoses [65,66,67]. These catecholamines can stimulate cancer cell proliferation and inhibit immune surveillance [68]. Multiple studies suggest a connection between adrenergic stimulation and the development of various cancers [65,66,69]. This association has led researchers to investigate the potential of yohimbine, which interacts with adrenergic receptors, as an anti-cancer agent.

G-protein-coupled receptors (GPCRs) represent a significant target for cancer therapies due to their extensive involvement in cancer progression [70,71]. As the largest protein superfamily in the human genome, GPCRs mediate crucial cancer-related events, including cell proliferation, metastasis, and angiogenesis. Their dysregulation is implicated in various cancer types, and targeting GPCRs signaling pathways has been associated with improved survival rates among cancer patients, underscoring the clinical relevance of these receptors as therapeutic targets [72]. Yohimbine, as an α2-adrenergic receptor antagonist, has emerged as a promising anticancer agent. Its anticancer activity stems from its ability to antagonize specific GPCRs involved in cancer development and progression. Studies have demonstrated yohimbine’s efficacy in inhibiting several cancer-related GPCRs, including α-2B adrenergic, dopamine D2B, and hydroxytryptamine receptors [23]. This multifaceted targeting of cancer-related GPCRs underscores yohimbine’s potential as a broad-spectrum anticancer agent.

Beyond its direct anticancer effects, yohimbine has also shown promise in treating benign prostatic hyperplasia (BPH). Studies have demonstrated its therapeutic potential against BPH by downregulating steroid 5α-reductase type 2, a key enzyme involved in the development of BPH [73]. This finding suggests that yohimbine may have broader therapeutic applications beyond cancer.

The study by Jabir et al. [74] also revealed significant findings regarding yohimbine’s potential as a multi-targeted anticancer agent. Researchers selected sixteen well-known anticancer targets and seven phytobioactive compounds, aiming to identify a compound that could effectively target multiple proteins involved in cancer therapy. Molecular docking results indicated that yohimbine exhibited the lowest docking scores among the tested compounds, ranging from −8.3 to −10.0 kcal/mol, which suggests a strong binding affinity to the studied protein targets. Furthermore, the protein-ligand interaction analysis demonstrated that yohimbine could feasibly bind to all selected targets, highlighting its multi-target interaction capability. The stability of the protein-ligand complexes was confirmed through molecular dynamics simulations, particularly with the three most scored targets: ERK2, PARP1, and PIK3α, which showed stable interactions with yohimbine throughout the simulation. Ultimately, the study concluded that yohimbine is the most potent compound among those evaluated, indicating its potential as an effective lead molecule against the studied target proteins in cancer therapy.

## 10. Myocardial Function and Cardiovascular Health

Yohimbine has demonstrated several positive effects on cardiovascular health, particularly in enhancing cardiac function and mitigating damage during stress conditions.

Research by Wang et al. [75] showed that yohimbine administration significantly improved cardiac function in LPS-challenged mice. LPS significantly decreased left ventricular ejection fraction and fractional shortening while increasing left ventricular end-diastolic pressure. Yohimbine pretreatment attenuated the LPS-induced decline in LVEF and FS and prevented the increase in LVEDP. Furthermore, it increased cardiac norepinephrine release in both control and LPS-treated mice. LPS alone did not significantly alter cardiac NE levels. The authors also found that YHB downregulated cardiac α2A-adrenergic receptor (α2A-AR) expression, particularly in the presence of LPS. Immunofluorescence analysis confirmed the presence of α2A-AR in cardiac sympathetic nerve presynaptic membranes. Additionally, YHB attenuated the LPS-induced increase in TNF-α, NO, and caspase 3/7 activity in cardiac tissue. Finally, the cardioprotective effects of yohimbine were abolished by the β1-AR antagonist atenolol, suggesting the involvement of β1-AR signaling in yohimbine’s mechanism of action.

Pretreatment with berberine and yohimbine demonstrated significant cardioprotective effects against lipopolysaccharide-induced myocardial dysfunction in mice in research by Wang et al. [76]. Both berberine and yohimbine, individually and in combination, improved left ventricular ejection fraction, fractional shortening, and early diastolic transmitral velocity, indicating a reduction in cardiac dysfunction. Furthermore, pretreatment with these compounds attenuated cardiac apoptosis and suppressed the overproduction of pro-inflammatory cytokines TNF-α and IL-1β, highlighting their anti-apoptotic and anti-inflammatory properties. The observed inhibition of I-κBα phosphorylation suggests a potential mechanism for these protective effects. Interestingly, the treatments did not significantly impact oxidative stress markers, suggesting their cardioprotective action is independent of this pathway. These findings suggest that yohimbine may be a promising therapeutic agents for preventing myocardial dysfunction during sepsis.

Lim et al. [77] demonstrated that Yohimbine exerts significant effects on platelet-derived growth factor-stimulated vascular smooth muscle cells. Yohimbine downregulated cell cycle regulatory proteins, including proliferating cell nuclear antigen, cyclin D1, cyclin-dependent kinase 4, and cyclin E, potentially via modulation of the transcription factor FOXO3a (Forkhead box O3). Furthermore, yohimbine dose-dependently decreased the phosphorylation of p-38 and mTOR, key components of proliferation and migration signaling pathways. A notable reduction in focal adhesion kinase phosphorylation at Y397 and Y925 sites, particularly Y925, which is associated with cell adhesion and migration, was also observed. Co-treatment of yohimbine with mTOR or p38 inhibitors further reduced VSMC migration and proliferation. These findings suggest that yohimbine may be a promising therapeutic candidate for preventing and treating cardiovascular diseases by targeting the FOXO3a and mTOR/p38/FAK signaling pathways.

Studies using human-induced pluripotent stem cell-derived cardiomyocytes have shown that yohimbine can directly alter electrophysiological properties, potentially impacting patients with atrioventricular conduction disorders [78]. This study investigated yohimbine’s effects on myocardial cells using isolated perfused chick embryonic hearts and cultured reaggregated cells. Yohimbine exhibited concentration-dependent effects on action potentials and ionic channels. At low concentrations (10⁻⁶ M), action potential duration was prolonged without significant changes in other parameters. However, higher concentrations (5 × 10⁻⁴ M) completely blocked fast Na⁺ channels, abolishing action potentials. This suggests greater sensitivity of fast Na⁺ channels to yohimbine compared to slow channels, which required even higher concentrations (10⁻^3^ M) for blockade. Yohimbine also displayed a dual effect on the isoproterenol-induced slow response, enhancing it at low doses but depressing or blocking it at high doses. Similarly, a positive inotropic effect was observed at low yohimbine concentrations, while higher concentrations caused a negative inotropic effect and eventual contractile blockade. These findings, consistent across both experimental models, suggest yohimbine acts like a local anesthetic on myocardial ionic channels, potentially by closing the inactivation gate of fast Na⁺ channels rather than direct channel occlusion.

The study by Chiu et al. [79] provides compelling evidence for the potential of yohimbine as a therapeutic agent for vascular proliferative diseases. Their research demonstrates that yohimbine effectively targets and suppresses the proliferation and migration of vascular smooth muscle cells (VSMCs) stimulated by platelet-derived growth factor-BB (PDGF-BB), a key player in vascular remodeling and disease. Yohimbine’s inhibitory effects are specifically mediated through the downregulation of the phospholipase C-γ1 signaling pathway. This pathway is crucial for transducing the effects of PDGF-BB on VSMCs, leading to increased proliferation and migration. The study’s findings are particularly noteworthy because yohimbine does not significantly affect other signaling pathways, such as ERK1/2, AKT, or p38 kinase, indicating a targeted and selective mechanism of action. This specificity reduces the likelihood of off-target effects and enhances its potential as a therapeutic agent. Further investigation revealed that yohimbine induces cell cycle arrest in the G0/G1 phase in VSMCs. This arrest effectively halts the progression of VSMCs through the cell cycle, thereby inhibiting their proliferation. The G0/G1 phase is a critical checkpoint in the cell cycle, and its disruption can contribute to uncontrolled cell growth and the development of vascular diseases. In addition to its anti-proliferative effects, yohimbine also exhibits anti-migratory properties. The study demonstrates that yohimbine reduces the expression of matrix metalloproteinases (MMP), specifically MMP-2 and MMP-9. MMPs are enzymes that play a crucial role in the breakdown of the extracellular matrix, facilitating cell migration and invasion. By reducing MMP expression, yohimbine further inhibits the progression of vascular proliferative diseases. The in vivo experiments conducted in a mouse model provide further support for yohimbine’s therapeutic potential. The study shows that yohimbine administration significantly reduces neointimal hyperplasia, a pathological thickening of the vascular wall that is a hallmark of various vascular proliferative diseases, including atherosclerosis and restenosis. This in vivo validation strengthens the case for yohimbine as a promising therapeutic strategy.

## 11. Cardiovascular Complications

While yohimbine has shown potential in the treatment of certain conditions, its effects on myocardial function and cardiovascular health have raised some concerns. Yohimbine is a potent stimulant of the sympathetic nervous system, acting as an antagonist of α_2_-adrenoceptors. This can lead to increased heart rate, blood pressure, and myocardial oxygen demand, potentially exacerbating underlying cardiovascular conditions [80].

The case reported by Song et al. highlighted the potential for yohimbine to induce significant cardiovascular complications, including Type II myocardial injury [81]. The 51-year old patient, with a past medical history of hypertension, hypercholesterolemia and obstructive sleep apnea, presented with palpitations, chest discomfort, and elevated troponin levels following yohimbine ingestion. Symptoms resolved with medical intervention, including nitroglycerin and aspirin administration. The mechanisms by which yohimbine may cause myocardial injury are multifaceted and include increased myocardial oxygen demand due to elevated blood pressure and heart rate, direct myocardial damage from increased norepinephrine release, and coronary vasoconstriction and spasm. This case underscores the importance of considering yohimbine as a potential cause of cardiac events in patients presenting with relevant symptoms. Given the widespread availability of yohimbine as an over-the-counter supplement, combined with its promotion for off-label uses such as erectile dysfunction, there is a critical need for increased public awareness regarding its potential cardiovascular risks. Clinicians should also be vigilant in inquiring about the use of over-the-counter supplements, including yohimbine when evaluating patients with unexplained cardiac symptoms. Further research is warranted to fully elucidate the mechanisms of yohimbine-induced cardiotoxicity and to develop appropriate risk mitigation strategies.

Gong et al. demonstrated that yohimbine has direct cardiotoxic effects on human-induced pluripotent stem cell-derived cardiomyocytes (hiPSC-CMs) [82]. After exposure to yohimbine, the frequency of spontaneous action potentials in hiPSC-CMs was inhibited, and the action potential duration was significantly prolonged in a dose-dependent manner. Specifically, the resting potential, threshold potential, amplitude, and maximal diastolic potential were all decreased while the APD50/APD90 ratios were prolonged. Furthermore, Yohimbine inhibited the amplitude of sodium (Na+) channel currents (INa) at low doses, with an IC50 value of 14.2 μM, and inhibited calcium (Ca++) channel currents (ICa) at higher doses, with an IC50 value of 139.7 μM. Notably, while the activation curves of both Na+ and Ca++ channels remained unaffected, the inactivation curves shifted leftward following treatment with yohimbine. These findings indicate that yohimbine directly alters the electrophysiological properties of hiPSC-CMs, independent of α2-adrenoceptor signaling, suggesting potential implications for its use in patients with atrioventricular conduction disorders.

## 12. Sport Performance

Yohimbine has also been historically used as a performance-enhancing supplement by athletes and bodybuilders, with claims of improving fat loss and increasing energy levels [83].

One of the newest studies, by Ballmann et al. [84], investigated the impact of yohimbine on high-intensity exercise performance in the morning. The researchers compared three different trial conditions: a placebo administered in the morning (PL-AM), yohimbine administered in the morning (YHM-AM), and a placebo administered in the afternoon (PM), serving as a control for diurnal variations in performance. The results demonstrated a significant diurnal variation in performance, with participants exhibiting lower power output and slower time to competition in the PL-AM trial compared to the PM trial (*p* = 0.010 and *p* = 0.007, respectively). However, yohimbine ingestion in the morning significantly improved both power output (*p* = 0.035) and TTC (*p* = 0.007) compared to the PL-AM trial, effectively mitigating the typical morning performance decline. Importantly, no significant differences were observed between the YHM-AM and PM trials for these performance metrics, suggesting that yohimbine effectively normalized morning performance to afternoon levels. Furthermore, yohimbine ingestion resulted in significantly lower post-exercise blood lactate levels compared to both PL-AM (*p* = 0.046) and PM (*p* = 0.001) trials, indicating improved metabolic efficiency during high-intensity exercise. Pre-exercise plasma hypoxanthine levels, a marker of metabolic stress, were significantly higher in the PM trial compared to PL-AM (*p* = 0.039), and while the YHM-AM trial showed a similar trend towards higher hypoxanthine levels compared to PL-AM, the difference was not statistically significant (*p* = 0.060). Subjective measures of energy and alertness were also significantly higher in the YHM-AM trial compared to PL-AM (*p* = 0.045 for both), aligning with the observed performance enhancements. Interestingly, despite the improvements in performance and metabolic markers, there were no significant differences in heart rate or rating of perceived exertion between the three trials, suggesting that the perceived effort remained consistent regardless of yohimbine ingestion. These findings collectively suggest that yohimbine may be a beneficial ergogenic aid for athletes seeking to improve their performance during morning training sessions.

Barnes et al. [22] examined the ergogenic effects of acute yohimbine hydrochloride supplementation on repeated supramaximal sprint performance. The study employed a placebo-controlled design, comparing the effects of yohimbine against a placebo on various performance and physiological markers. The results revealed significant improvements in several key performance indicators following yohimbine ingestion. Specifically, participants in the yohimbine group demonstrated significantly higher mean power output (*p* < 0.001; η^2^ = 0.024) and total work (*p* < 0.001; η^2^ = 0.061) compared to the placebo group, indicating a substantial enhancement in sprint performance. Moreover, the fatigue index, a measure of the decline in power output over repeated sprints, was significantly lower in the yohimbine group (*p* < 0.001; η^2^ = 0.054), suggesting that yohimbine helped maintain power output across multiple sprints. This improvement in fatigue resistance is a crucial factor in high-intensity intermittent exercise performance. The study also observed significant physiological changes associated with yohimbine supplementation. Blood lactate levels post-exercise were significantly reduced in the yohimbine group (*p* < 0.001; d = 1.26), suggesting either improved lactate clearance or reduced lactate production during the sprints. This finding further supports the performance-enhancing effects of yohimbine, as lower lactate levels can contribute to delayed fatigue. Additionally, yohimbine supplementation led to significantly higher concentrations of epinephrine in both pre- and post-exercise (*p* < 0.001; η^2^ = 0.225), indicating an increased sympathetic response. While norepinephrine levels also increased over time, the treatment did not significantly affect this response (*p* < 0.001; η^2^ = 0.227). Heart rate was significantly higher in the yohimbine group (*p* < 0.001; η^2^ = 0.046), likely reflecting the increased sympathetic activity. However, there were no significant differences in the rate of perceived exertion between the yohimbine and placebo groups (*p* = 0.539; η^2^ < 0.001), suggesting that the perceived effort remained similar despite the enhanced performance. These findings collectively suggest that acute yohimbine ingestion can positively influence repeated supramaximal sprint performance, potentially through mechanisms involving improved lactate metabolism and increased sympathetic nervous system activation.

The study by Williams et al. [85] investigating the effects of acute yohimbine hydrochloride ingestion on bench press performance in resistance-trained males found that yohimbine significantly improved muscular strength-endurance, as evidenced by a greater number of repetitions to failure (*p* = 0.002; d = 0.82). However, it did not significantly affect mean power (*p* = 0.472; d = 0.16) or mean velocity (*p* = 0.297; d = 0.25), indicating that while strength endurance improved, explosive performance metrics remained unchanged. Participants also reported increased motivation (*p* = 0.011; d = 0.64) and energy levels (*p* < 0.001; d = 1.27), and reduced fatigue (*p* < 0.001; d = 1.65) after yohimbine ingestion. These findings suggest that yohimbine may enhance psychological factors contributing to improved performance in resistance training, specifically by combating fatigue and increasing endurance, but not explosive power, during resistance exercises.

A study investigating yohimbine’s ergogenic effects in healthy volunteers found that a single 5 mg oral dose significantly improved several exercise parameters during cycling, including effort exerted, calories burned, distance covered, speed, and total exercise time (*p* < 0.05). Yohimbine also significantly increased maximal oxygen uptake (VO2 max) and the velocity at VO2 max (*p* < 0.05), indicating enhanced aerobic capacity and oxygen utilization during exercise. Positive effects were also observed on heart rate metrics, further supporting yohimbine’s ergogenic potential. These findings suggest yohimbine can enhance cycling performance by improving both physical output and oxygen consumption. It is worth noting that while this study highlights yohimbine’s benefits for endurance exercise, other research mentioned in your document shows different effects on strength-based activities like bench presses and repeated sprints, suggesting that yohimbine’s impact on performance may vary depending on the exercise modality.

## 13. Behavioral Sensitivity

Yohimbine’s impact on behavior is complex and multifaceted, influenced by factors such as dosage, individual differences, and the specific behavior being measured. It can induce anxiety-like responses in both humans and animals [86], potentially by increasing norepinephrine levels in the brain. This anxiogenic effect has led to its use in some research settings as a pharmacological stressor to study the impact of stress on various behaviors.

Fricke et al. [87] investigated how pharmacological modulation of key stress regulators impacted task-based approach-avoidance conflict behavior in a cohort of healthy participants. Their study involved a group of 96 individuals (randomly assigned to receive either 20 mg of hydrocortisone, 20 mg of yohimbine, a combination of both, or a placebo prior to completing a task that examined foraging behavior under conditions of potential predation in a fully crossed double-blind between-subjects experimental design), wherein the administration of 20 mg of yohimbine effectively stimulated the noradrenergic system, as demonstrated by elevated alpha-amylase activity, indicating its impact on physiological stress indicators. Yohimbine specifically impacted risky foraging latency under predation, altering participants’ behavior in the approach-avoidance task, particularly in their response to potential threats. Furthermore, yohimbine interacted with the personality trait of anger, affecting foraging speed, with participants who had not taken yohimbine approaching foraging faster when further from the threat. Gender differences were also observed, with males generally outperforming females in token collection and capture avoidance, potentially influenced by yohimbine’s interaction with endogenous testosterone levels. However, despite these specific effects, no main effect of yohimbine on overall approach-avoidance behavior was found when considering the combined effects of hydrocortisone and yohimbine, suggesting that while yohimbine influenced certain aspects of behavior, it did not replicate broader stress effects observed in other studies. These findings highlight the need for further research into yohimbine’s role in approach-avoidance conflicts, particularly concerning its interactions with gender and personality traits, to better understand the mechanisms underlying stress-related behaviors.

Later on Yohimbine, Munster et al. [88] studied whether yohimbine acts as an acute pharmacological stressor influencing decision-making in male rats. By blocking receptors that typically inhibit norepinephrine release, yohimbine increases arousal and stress responses. Systemic administration of yohimbine significantly increased preference for a risky/large reward lever over a certain/small reward, suggesting that it enhances risk-taking in reward-based decisions. Interestingly, corticosterone, another stress-related hormone, did not produce a similar effect, indicating that yohimbine possesses unique properties that specifically promote risky choices. Further investigation revealed that blocking dopamine D1 receptors in the dorsal prelimbic cortex reduced the yohimbine-induced preference for the risky option, suggesting that activating these receptors is crucial for yohimbine’s influence on risk-taking. These findings provide causal evidence that stimulating PL D1 receptors may be a neurochemical mechanism by which yohimbine, and potentially other stressors, affect decision-making processes related to risk. Furthermore, yohimbine has been investigated for its potential effects on impulsivity and other aspects of behavioral control. Studies by Herman et al. [26] have explored its role in conditions like ADHD and PTSD, where impulsivity is a prominent feature. This study examined the effects of yohimbine-induced arousal on impulsivity in 43 healthy volunteers. Participants received either yohimbine or a placebo and completed various impulsivity measures while their blood pressure and heart rate were monitored. Yohimbine significantly increased blood pressure, which was linked to changes in impulsivity. The yohimbine group showed improved response inhibition on the Stop Signal Task, suggesting decreased motor impulsivity. Increased blood pressure after yohimbine was also associated with more far-sighted decisions on the Delay Discounting Task, indicating lower temporal impulsivity. However, higher blood pressure was linked to reduced information gathering on the Information Sampling Task, suggesting increased reflection impulsivity. No significant effects were observed on other impulsivity measures, heart rate, or self-perceived arousal. While yohimbine increased negative affect, it did not alter positive affect.

Although yohimbine is not a first-line treatment for depression, it has shown some promise as an adjunctive therapy or for specific subtypes of depression. Its proposed antidepressant mechanism primarily revolves around its antagonism of α_2_-adrenergic receptors [83,89,90,91]. By blocking these receptors, yohimbine increases noradrenaline levels in the synaptic cleft, which can counteract the reduced noradrenergic activity often associated with depression [89,91].

Some studies suggest that yohimbine may be particularly effective in atypical depression, a subtype characterized by increased appetite, hypersomnia, and mood reactivity [83]. However, the evidence for its efficacy in this subtype is still limited and requires further investigation.

Yohimbine’s interaction with serotonergic receptors, specifically 5-HT_1_A, 5-HT_1_B, and 5-HT_1_D receptors, may also contribute to its antidepressant effects [91]. However, the precise nature of these interactions and their clinical significance remains to be fully elucidated.

It is important to note that yohimbine’s use as an antidepressant is not without limitations. Its potential for side effects, including anxiety, nervousness, and insomnia, can be problematic for some individuals [92]. Furthermore, its interaction with other medications, such as antidepressants and antihypertensives, requires careful consideration [91].

Emerging evidence suggests that the combination of yohimbine with traditional antidepressants, such as the selective serotonin reuptake inhibitor fluoxetine, may hold promise in accelerating the antidepressant response in clinical settings [90]. While the underlying mechanisms are not fully understood, the synergistic effects of these compounds are thought to involve the modulation of both noradrenergic and serotonergic neurotransmitter systems. Yohimbine, by blocking alpha-2 adrenergic receptors, can increase norepinephrine availability, while fluoxetine enhances serotonin levels [89,90,91]. This multimodal approach may help address the complex pathophysiology of depression more effectively, potentially leading to faster symptom improvement and improved clinical outcomes for patients. However, further research is needed to fully elucidate the optimal dosing, timing, and patient populations that may benefit most from this combination therapy approach.

## 14. Effects of Yohimbine Depending on Concentration

Yohimbine presents a complex interplay of beneficial and detrimental effects, highly contingent upon its concentration—at lower doses demonstrating promising therapeutic potential. For instance, a 5 mg/kg/day dose significantly reduced hepatic sulfate levels to control values and induced rhodanese expression, an enzyme crucial for sulfur metabolism [57]. This higher dose also demonstrated a reduction in lipid peroxidation, indicating a protective effect against oxidative stress. A lower dose of 2 mg/kg/day also showed a trend towards decreased sulfate levels, although this effect was not statistically significant. Interestingly, the 2 mg/kg/day dose restored cystathionine γ-lyase activity to control levels, an effect not observed with the higher dose. These findings suggest that yohimbine’s effects on sulfur metabolism and oxidative stress are dose-dependent, with different mechanisms potentially activated at varying concentrations.

Furthermore, within the concentration range of 0.1 to 1 µM, yohimbine enhances synaptic activity by increasing the overflow of neurotransmitters such as noradrenaline and serotonin [93,94]. However, this seemingly beneficial profile shifts dramatically at higher concentrations—above 10 µM can counterintuitively diminish its efficacy by reducing neurotransmitter overflow below baseline levels, thereby inhibiting synaptic transmission and has been shown to induce neuronal death through apoptotic pathways, indicating a clear biphasic dose-response relationship.

Experimental studies utilizing a wider range of doses (0.5 to 4.5 mg/kg) in rats further confirm yohimbine’s dose-dependent influence on thermal, locomotor, and cardiovascular responses [95]. At low doses (below 1 mg/kg), it primarily functions as an alpha2-adrenoblocker, inhibiting receptors that suppress neurotransmitter release. Moderate doses (1–2 mg/kg) are sufficient to counteract general anesthesia in rats. However, at higher doses (exceeding 1 mg/kg), yohimbine’s mechanism shifts to that of a 5-HT1A agonist, activating serotonin receptors and potentially influencing physiological parameters such as heart rate and body temperature. These higher doses also trigger the release of stress-related hormones like ACTH and corticosterone.

Elevated yohimbine levels, such as 8000 µg/L (the highest ever reported in cases of yohimbine intoxication), have been associated with severe cardiovascular complications, including type II myocardial injury and acute, potentially fatal intoxication [1,81]. This concentration-dependent dichotomy underscores the critical need for precise dosage control and careful consideration of potential risks when utilizing yohimbine in clinical or supplemental applications.

## 15. Conclusions

Yohimbine demonstrates therapeutic potential in various conditions like erectile dysfunction, weight loss, metabolic disorders, myocardial dysfunction, depression, inflammatory disorders and cancer due to its interaction with multiple monoaminergic receptors and its stimulant properties from blocking α2-adrenergic receptors. While its exact action mechanism is unclear, it exhibits drug-like properties. Despite toxicological concerns at higher doses, further research, including preclinical and clinical trials (Table 1), is needed to fully assess its efficacy and safety.

Exploring its potential as adjunct therapy, improving bioavailability, and reducing toxicity through methods like neo-synthesis or nanotechnology could enhance its clinical value. Further research, especially clinical trials, is crucial to establish yohimbine as a reliable pharmaceutical agent and to understand its pharmacokinetics, molecular mechanisms, and safety profile for human use. This underscores the importance of evaluating traditional remedies for modern medical applications.

## Figures and Tables

**Table 1:** Yohimbine—clinical trials.

NCT Number	Study Title	Study URL	Brief Summary	Sex	Phases	Completion Date
NCT0159321 5	Randomized Study of Yohimbine Treatment for Type 2 Diabetes Patients Carrying a Specific Genetic Risk Variant	https://clinicaltrials.gov/study/NCT01593215 (accessed on 18 November 2024)	The investigators have recently discovered a genetic variant in an adrenergic receptor that leads to increased risk for type 2 diabetes. The investigators have also seen that blockers of that receptor improves impaired insulin secretion in animals. The investigators will now test the blocker in patients with type 2 diabetes with or without the risk variant in an effort to make diabetes treatment more individualized.	ALL	PHASE2	2014-10
NCT0103197 9	Prolonged Exposure for Post Traumatic Stress Disorder (PTSD) With/Without Yohimbine	https://clinicaltrials.gov/study/NCT01031979 (accessed on 18 November 2024)	The proposed study has three distinct but related research objectives. The first goal is to measure physiological correlates of successful treatment with Prolonged Exposure (PE) therapy for posttraumatic stress disorder (PTSD) in veterans of the Iraq and Afghanistan wars. Individuals with PTSD often experience elevated heart rates and other objectively measurable signs of anxiety when confronted with safe situations that remind them of past dangerous situations. We will measure physiological responses and compare the outcomes to patient’s self-reported subjective accounts of symptom improvement on traditional measures of PTSD. Developing a way to measure objective gains in symptoms improvement may help researchers who are studying ways to improve PTSD treatment. The second goal of the study is to investigate if yohimbine, a drug found to promote a specific type of learning, will improve treatment outcomes for veterans in PTSD treatment. The third goal is to investigate if ability to get used to loud startling audio tones correlates to baseline PTSD pathology and treatment outcomes for PE. This goal represents an important step forward in understanding characteristics of heritable traits that are related PTSD. It is significant because such research may one day lead to the development of individual responder policies that will assist patients by individualizing treatment plans based on personal characteristics.	MALE	PHASE2	2015-07-07
NCT0007871 5	Rapid Antidepressant Effects of Yohimbine in Major Depression	https://clinicaltrials.gov/study/NCT00078715 (accessed on 18 November 2024)	This study examines if Yohimbine, when given during the sleep cycle, will improve symptoms of depression within a matter of hours.	ALL	PHASE2	2009-08
NCT0053500 2	The Effect of Yohimbine on Cocaine Cue Reactivity	https://clinicaltrials.gov/study/NCT00535002 (accessed on 18 November 2024)	Purpose: This study will examine whether the drug yohimbine, given at a specific time during the sleep cycle, produces chemical changes in the brain similar to those that occur with sleep deprivation. It will also see if yohimbine can induce rapid (next day) antidepressant effects in patients with major depression. Total sleep deprivation for 36 h improves mood in most patients with major depression in a matter of hours, but the response is usually short-lived. Understanding the chemical changes that occur in the body during sleep deprivation may help in the development of a rapidly acting antidepressant.Patients with major depressive disorder between 18 and 65 years of age may be eligible for this study. Candidates are screened with a medical and psychiatric history, physical examination, electrocardiogram, and blood and urine tests. Participants are hospitalized at the NIH Clinical Center for the study, as follows: Drug- free period: Patients are tapered off their anti- depression medications and remain drug-free for 1 week before beginning study phase 1. Study phase 1: Patients undergo sleep deprivation for 36 h. Those whose depression improves with sleep deprivation initially and then worsens continue to phase 2. The day after sleep deprivation, patients undergo a lumbar puncture (spinal tap). For this test, a local anesthetic is given and a needle is inserted in the space between the bones in the lower back where the cerebrospinal fluid circulates below the spinal cord. A small amount of fluid is collected through the needle. Study phase 2: Patients spend 1 night in the sleep lab. A catheter (plastic tube) is placed in a vein in each arm-one to give yohimbine and the other to draw blood samples. A small monitor cuff is placed on a finger to measure the patient’s blood pressure and blood oxygen levels during the night. While asleep, the patient receives a dose of yohimbine or placebo, given over 3 min. A lumbar puncture is done the following morning. Patients receive no medications for 6 days, and then the sleep lab procedure is repeated. Patients who received yohimbine in the previous experiment are switched to placebo, and those who were given placebo are switched to yohimbine. Stress and cues reminiscent of cocaine use promote craving and relapse in cocaine dependent individuals. In addition, there appears to be gender differences in determinants of relapse to drug use following abstinence in cocaine-dependent individuals. Therefore the purpose of the present study is to study the role of hormonal status on the response to cocaine-related cues with or without stress in cocaine-dependent women and men.	ALL	PHASE2	2012-08
NCT0095888 0	Yohimbine to Enhance Cognitive Behavioral Therapy (CBT) for Social Anxiety	https://clinicaltrials.gov/study/NCT00958880 (accessed on 18 November 2024)	The purpose of this study is to investigate the utility of Yohimbine hydrochloride for facilitating fear extinction in a sample of patients with social phobia who will be treated with CBT.	ALL	PHASE3	2013-01
NCT0060590 4	Modulation of Pharmacologically Induced Alcohol Craving in Recently Detoxified Alcoholics	https://clinicaltrials.gov/study/NCT00605904 (accessed on 18 November 2024)	This study will determine if acamprosate, a drug approved to treat alcoholism, decreases alcohol cravings in alcohol-dependent subjects following infusions of yohimbine and mCPP. Yohimbine causes anxiety and may provoke a desire for alcohol; mCPP induces a feeling of having had a few drinks, which often creates a desire for more drinks. If acamprosate can prevent a craving following these stimuli, then the effectiveness of new experimental drugs for treating alcoholism can be tested for their ability to block yohimbine or mCPP-induced cravings. This type of investigation would be less expensive and less time-consuming than conducting clinical trials with alcohol- dependent people. People between 21 and 65 years of age who are alcohol-dependent and have been drinking regularly for at least 1 month before entering the study may be eligible to participate. Participants are admitted to the NIH Clinical Center for about 35 days, during which time they are asked to participate in an alcohol treatment program. They may request passes to leave the hospital during the day but must return overnight. Upon return to the hospital, subjects are required to take a breathalyzer test for alcohol and urine screen for drug use. Participants found to have used drugs or consumed alcohol while away from the hospital are terminated from the study. Participants are randomly assigned to take acamprosate or placebo pills three times a day for about 2 weeks. They are then given three intravenous (through a vein) infusions, 5 to 7 days apart, each containing either yohimbine, mCPP or placebo. The drugs are infused for 20 min following a 1-h infusion of saline (salt water). Subjects complete two questionnaires—an alcohol urge questionnaire to assess the desire for alcohol and a PASS rating scale to assess anxiety—several times during the study and during the infusions....	ALL	PHASE2	2011-03
NCT0224370 9	Mifepristone for the Prevention of Relapses of Alcohol Drinking	https://clinicaltrials.gov/study/NCT02243709 (accessed on 18 November 2024)	The goal of this study is to determine if, under stress, alcohol drinking is reduced using mifepristone	ALL	PHASE1 PHASE2	2021-12-21

## Data Availability

No new data were created or analyzed in this study. Data sharing is not applicable to this article.
